# Multimodal computational modeling of EEG and artistic painting for exploring the stress-relief mechanism of urban green spaces

**DOI:** 10.3389/fpsyg.2025.1547947

**Published:** 2025-10-07

**Authors:** Tang Bei

**Affiliations:** Suzhou University College of Arts and Design, Suzhou, Anhui, China

**Keywords:** clip, attention fusion, dynamic brushwork, real-time painting, AI art generation

## Abstract

**Introduction:**

Multimodal learning has recently opened new possibilities for integrating semantic understanding into creative domains, and models like Contrastive Language-Image Pretraining (CLIP) provide a compelling foundation for bridging text-image relationships in artistic applications. However, while CLIP demonstrates exceptional capabilities in image-text alignment, its application in dynamic, creative tasks such as freehand brushwork painting remains underexplored. Traditional methods for generating artwork using neural networks often rely on static image generation techniques, which struggle to capture the fluidity and dynamism of brushstrokes in real-time creative processes. These approaches frequently lack the interpretive flexibility required to respond to real-time textual prompts with spontaneous, expressive outcomes.

**Methods:**

To address this, we propose ArtCLIP, a novel framework that integrates CLIP with an attention fusion mechanism to facilitate dynamic freehand brushwork painting. Our method utilizes CLIP's ability to interpret textual descriptions and visual cues in tandem with an attention-based fusion model, which enables the system to modulate brushstrokes responsively and adjust painting styles dynamically based on evolving inputs.

**Results and discussion:**

We conduct extensive experiments demonstrating that ArtCLIP achieves significant improvements in real-time artistic rendering tasks compared to baseline models. The results show enhanced adaptability to varying artistic styles and better alignment with descriptive prompts, offering a promising avenue for digital art creation. By enabling semantically driven and stylistically controllable painting generation, our approach contributes to a more interpretable and interactive form of AI-assisted creativity.

## 1 Introduction

Creating dynamic freehand brushwork painting using AI has become an increasingly important area of research due to its potential to bridge the gap between human creativity and computational assistance (Feng, 2023). The challenge of generating freehand-style artwork dynamically, as in traditional brush painting, requires sophisticated modeling of both visual and semantic content. Not only does this task involve capturing the fluidity and expressiveness of brushstrokes, but it also requires an understanding of the contextual and compositional elements of the artwork (Zou, 2023). Moreover, developing such systems not only enhances artistic creativity but also opens new avenues for interactive AI applications in creative industries, from digital art creation to education and entertainment (Gui, 2022).

In the early stages of computer-generated artwork, symbolic AI and knowledge representation techniques were the dominant methods for creating visual compositions (Gui, 2022). These approaches relied heavily on predefined rules and logical structures, where visual elements like lines, shapes, colors, and textures were encoded into symbolic representations. For example, an artist might describe a circle as a geometric form with specific parameters such as radius, color, and position, and the AI would use these symbols to recreate the visual (Fan, 2024). Rule-based logic allowed computers to assemble these symbols into simple forms of visual art, often resembling abstract or geometric designs (Wu, 2023). However, the inherent rigidity of symbolic systems made it challenging to capture the nuances of human creativity, particularly in art forms that required fluidity and subtlety, such as freehand brushwork or complex abstract painting. One of the core limitations of symbolic AI in the realm of art generation was its dependence on handcrafted rules and expert knowledge. For the system to function effectively, it required a deep understanding of both the visual elements it was working with and the aesthetic principles behind them (Zheng and Zhao, 2023). This dependency created bottlenecks in artistic diversity and expressiveness. While symbolic AI could generate artwork based on logical constructs, it struggled to replicate the spontaneity and improvisational nature of human art (Sun, 2021). The structured rules that governed its output lacked the flexibility to evolve organically, which is often crucial in the creative process (Li et al., 2023). Furthermore, the reliance on predefined parameters made it difficult for these systems to generalize across various artistic styles, limiting their application to niche areas of visual representation (Zhang, 2024). For example, interpreting and generating abstract art, which often defies traditional structures, was especially difficult for these systems, as they were not equipped to handle the interpretative and emotional layers that such art often entails.

To address the shortcomings of symbolic AI, researchers turned to data-driven methods and machine learning (ML) as an alternative approach to computational creativity (Chen, 2020). Unlike symbolic AI, which was constrained by rigid rules, machine learning models could learn from data, allowing them to infer patterns and relationships between visual elements without relying on explicit programming of these connections (Wang and Ni, 2023). By training models on large datasets of images, accompanied by labels that described their content, machine learning systems were able to “understand” and generate artwork in a more flexible and adaptable manner (Li and Chen, 2021). For example, a machine learning model trained on thousands of landscape paintings could begin to synthesize new landscapes by identifying common visual themes, such as the shape of mountains or the colors of a sunset. This shift toward machine learning brought a significant improvement in the ability to create artwork with greater variability and less reliance on rigid structures (Gao and Xu, 2021). However, the fluidity and subtle imperfections characteristic of dynamic brushwork remained a challenge. Machine learning models, particularly early iterations, often excelled at replicating structured patterns and repeating learned motifs, but they struggled to generate artwork with the intentionality and depth of human-created paintings (Peng et al., 2024). The brushstrokes produced by these models, for instance, often lacked the nuanced control seen in traditional freehand painting, where the artist's intuition and personal touch play crucial roles. Moreover, machine learning models still faced challenges related to the data they were trained on (Andrade 2022). For a model to learn a specific artistic style, it required a significant amount of annotated data–something that could be both labor-intensive and difficult to obtain, especially for niche or abstract art styles. In cases where such data was sparse, the models often failed to generalize, producing results that were either too simplistic or too disconnected from the intended style (Tang et al., 2017).

The advent of deep learning, particularly with models like Contrastive Language-Image Pretraining (CLIP), has revolutionized the field of computer-generated art (Huang et al., 2022). Unlike traditional machine learning models, which often required vast amounts of domain-specific data to generate relevant art, CLIP introduced a new paradigm by learning from both images and text in a more holistic manner (Fu et al., 2021). CLIP was designed to understand visual concepts in relation to natural language, making it exceptionally versatile. Instead of relying solely on visual data, CLIP could process text-based prompts and generate images that were semantically aligned with the descriptions. This ability to link language with visual representation opened up new possibilities in the realm of creative AI, particularly in generating diverse and semantically rich artworks. CLIP's ability to fuse language and image understanding allows for far more nuanced and contextually relevant creations than previous AI systems (Machuca et al., 2023). By leveraging large-scale datasets that contain both images and corresponding textual descriptions, CLIP can generate artwork that not only looks aesthetically pleasing but also carries deep semantic meaning. For instance, a prompt like “a surreal landscape with melting clocks” would guide the AI to produce a scene inspired by Salvador Dalí's iconic style, blending both the surrealistic visual motifs and the conceptual underpinnings of the description. Moreover, deep learning architectures equipped with attention mechanisms further enhance the model's capability to manage complex patterns and layers of abstraction, allowing for more expressive and intricate art generation. This is particularly useful when dealing with abstract or multi-layered artistic concepts that require a deeper understanding of both form and meaning (Zeng et al., 2024). However, despite these advancements, challenges remain in achieving precise control over individual brushstrokes. While deep learning models can generate highly expressive and dynamic artwork, they still struggle to maintain coherence over extended artistic processes, such as in freehand painting. The fluidity required to produce continuous, smooth transitions between strokes, which is central to traditional painting techniques, is not easily replicated by even the most advanced AI systems (Qi et al., 2024). The complexity of this task lies in the fact that human painters often rely on intuition and subtle motor skills, qualities that are difficult for current deep learning models to emulate. Therefore, while CLIP and similar models represent a significant step forward in the domain of creative AI, further research is needed to enhance the fine-grained control required for truly lifelike brushwork in computational art.

Recent EEG-based research has demonstrated the method's ability to capture not only emotional states but also perceptual adaptation processes in response to environmental stimuli. Pourghorban et al. (2025b) showed that short-term thermal adaptation and alliesthesia are reflected in EEG dynamics, particularly through modulations in alpha and theta bands during rapid transitions and steady-state exposure. Their findings support the notion that EEG can effectively trace sensory-affective integration as the brain adjusts to new thermal environments. Similarly, their fine-grained EEG analysis of thermal perception emphasized the capacity of EEG to detect localized neural responses associated with comfort evaluation and perceptual transitions (Pourghorban et al., 2025a). These insights are directly relevant to our study, as they reinforce the interpretation that urban green space exposure may trigger not only emotional responses but also fine-tuned perceptual adaptation across sensory modalities. Thus, EEG responses in our data may reflect a broader alliesthetic process involving multisensory integration, contributing to the observed reduction in perceived stress.

The feasibility of real-time stress evaluation has been demonstrated through EEG-based signal processing. For example, (Purnamasari and Fernandya 2019) proposed a K-nearest neighbor approach for detecting stress states and guiding meditation interventions in real-time applications. Similarly, Nirabi et al. (2021) employed machine learning techniques to classify stress levels from EEG features, highlighting the potential for signal-driven stress modeling. Katmah et al. (2021) further provided a comprehensive review of EEG-based stress detection methods, emphasizing physiological signal patterns associated with mental strain. While these studies focus primarily on real-time classification and stress quantification, they often lack integration with expressive or perceptual outputs. In contrast, this study goes beyond detection by embedding EEG-derived stress signals into an interactive generative framework that enables artistic expression of neurophysiological states. This positions freehand painting not only as a passive reflection of affect but as an intervention mechanism tightly coupled with individual stress responses, particularly in natural green space contexts.

To address the limitations of the previous methods, we propose a novel approach that utilizes CLIP's ability to map both visual and linguistic information, combined with attention fusion mechanisms to create dynamic freehand brushwork painting. This approach leverages attention fusion to integrate semantic guidance with the precision control needed for individual brushstrokes, thereby overcoming the limitations of symbolic AI's rigidity and machine learning's lack of expressiveness. By allowing the model to attend dynamically to both global composition and fine-grained details, we enhance the system's ability to maintain coherence in complex, freehand-style paintings. Our method reduces reliance on large datasets by utilizing the pre-trained capabilities of CLIP, making it more adaptable to various artistic styles without extensive manual annotation.

- Our method introduces an innovative attention fusion module that dynamically combines semantic and visual cues to generate coherent and expressive brushstrokes.
- It excels in multi-scenario adaptability, offering high efficiency and generalization across different artistic styles, while also being able to control the expressiveness of individual strokes.
- Experimentally, our approach demonstrates superior performance in generating dynamic freehand brushwork, maintaining both visual coherence and artistic fluidity across diverse tasks.

## 2 Related work

### 2.1 Vision-language models in artistic creation

The intersection of vision-language models and artistic creation has become a prominent research area, particularly following the success of models like Contrastive Language-Image Pretraining (CLIP). CLIP's ability to align images and textual descriptions in a shared embedding space has opened up new possibilities for automating and augmenting artistic processes (Zhang et al., 2024). In this context, CLIP's bidirectional image-text representation is leveraged to generate art that responds to or is guided by text prompts. Various methods explore the integration of vision-language models in creative domains, enabling dynamic interactions between user inputs and model-generated responses. In particular, generative models based on CLIP have been used to transform textual descriptions into corresponding visual outputs. Researchers have focused on generating stylized images, synthesizing artwork, and even guiding artists in real-time by matching brushstrokes with semantic meanings derived from text prompts. CLIP provides a mechanism for aligning user-defined input with high-level artistic goals extracted from language. This alignment enables not only the generation of images but also the dynamic modification of artwork as it is being created, responding to ongoing textual or visual inputs (Wang et al., 2019b). Another area of exploration within this domain is how vision-language models can assist with freehand drawing and painting, where user input takes the form of strokes or gestures. CLIP can be integrated into neural networks that analyze and interpret brushwork, enabling real-time adjustments to artistic creations based on predefined goals (Chen et al., 2024). These models learn to associate the structural aspects of freehand drawing, such as lines and textures, with descriptive language, allowing artists to specify their intended output through a combination of drawing and text input. This has led to research focusing on interactive co-creation systems where human artists and machine intelligence collaborate dynamically.

### 2.2 Painting as an intervention medium: EEG-based modeling perspective

Attention mechanisms have played a significant role in recent advancements in neural networks, particularly in the domain of sequence-to-sequence tasks such as translation, text generation, and image captioning. In the context of artistic creation, attention mechanisms are used to capture both local and global dependencies in an image, which is essential for generating coherent and visually appealing artworks. By directing focus toward specific areas of an image, attention-based models allow for finer control over the generation process, enabling nuanced adjustments in real-time based on the artistic intent of the user (Li et al., 2024). Within this framework, attention fusion has emerged as a method to combine multiple types of inputs, such as freehand brushwork and textual descriptions, into a unified model. This fusion enables the system to consider the importance of both the visual and linguistic aspects of the input. For instance, a model could focus on the semantics of a descriptive prompt while simultaneously attending to the precision and dynamics of user-generated brushstrokes. Attention fusion allows for a more natural blending of these two modalities, leading to more cohesive and contextually relevant artistic outputs (Wang et al., 2019a). In dynamic freehand brushwork creation, attention mechanisms allow the system to respond to subtle changes in the artist's input, enabling features such as stroke refinement or texture enhancement in real time. For example, as an artist modifies a brushstroke, the attention Adaptive Stroke Renderingan prioritize those changes that align most closely with the textually defined artistic goal, effectively creating a feedback loop where the system's attention adapts to both the visual input and linguistic constraints. This leads to more interactive and fluid creative processes, where the artist's freehand inputs are dynamically modulated by the attention-driven response from the model (Xu et al., 2024).

### 2.3 Interactive co-creation with neural networks

The integration of neural networks into the creative process has shifted from passive generation to interactive co-creation, where human and machine collaborate in real time. This paradigm shift emphasizes the role of the human user as a co-creator, guiding the model through incremental inputs such as text, brushstrokes, or other forms of artistic expression. In such systems, the neural network adapts to user input, enabling a back-and-forth dialogue between the artist and the machine, where both parties contribute to the final artwork (Atzori et al., 2016). Interactive co-creation systems typically rely on reinforcement learning or other adaptive techniques to fine-tune the model's outputs based on ongoing user interactions. These systems must balance user control with the model's creative freedom, ensuring that the final output reflects the artist's vision while still benefiting from the computational power and creativity of the neural network. In dynamic freehand brushwork painting, this interaction is often mediated by vision-language models like CLIP, which allow the artist to steer the creative process using both natural language and visual input (Wang et al., 2016). One key aspect of interactive co-creation is the system's ability to interpret and respond to subtle cues from the artist, such as slight variations in brush pressure or speed, as well as changes in the descriptive prompts guiding the artwork. This requires real-time analysis of both the visual and linguistic data streams, as well as the capacity to dynamically fuse these inputs to generate art that is responsive and coherent (Hu et al., 2024). By integrating attention mechanisms, neural networks can focus on the most relevant aspects of the input, ensuring that the system's responses are aligned with the artist's evolving vision. This allows for a more engaging and iterative creative process, where the machine actively contributes to the artwork while adapting to the artist's ongoing input.

## 3 Method

### 3.1 Overview of multimodal freehand painting pipeline

**Figure 1:**
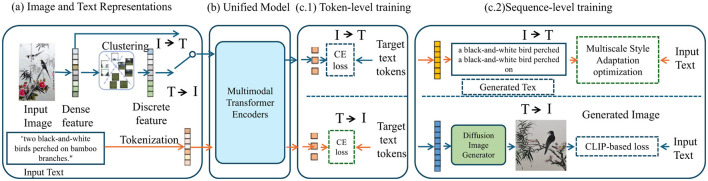
The proposed ArtCLIP freehand painting creation framework. **(a)** Image and Text Representations: Input images and text are processed into dense and discrete features. Images undergo clustering to obtain discrete features, while text is tokenized. **(b)** Unified model: a multimodal transformer encoder is trained for two tasks: image-to-text (I → T) and text-to-image (T → I), optimized using cross-entropy loss. **(c)** Sequence-level training: higher-level training includes generating images from text and generating text from images, using multiscale style adaptation optimization and CLIP-based loss, respectively.

The rapid advancement in generative models has increasingly facilitated creative processes across various forms of artistic expression. Particularly, the intersection of computational techniques and traditional art forms such as freehand brushwork painting has opened up new avenues for both artistic exploration and automation. In this work, we propose a novel approach to synthesizing multimodal freehand brushwork paintings, combining text, visual, and gestural inputs to guide the creative process. Our method builds upon the current state-of-the-art in multimodal generative models, integrating text-to-image frameworks with additional modalities, such as stroke-based input, to enable the generation of brushwork paintings that exhibit fine stylistic detail and artistic coherence. This section provides an overview of our methodology for generating freehand brushwork paintings using multimodal inputs. In particular, we explore how traditional brushstroke techniques can be replicated and enhanced through a combination of deep learning techniques and multimodal data fusion. The subsequent sections will delve into the detailed components of our method, outlining the preliminary concepts behind the model and the innovations that drive the new painting generation pipeline (as shown in Figure 1).

In Section 3.2, this study lays out the key preliminaries necessary for understanding the construction of our multimodal freehand brushwork painting generation model. This includes a discussion on how this study formalizes the generation process and the core elements that guide our multimodal integration. This study examines the role of text as a narrative-driven input, image features as the basis for visual style, and hand-drawn strokes or gestures that provide intricate control over the final output. These elements are fused in a coherent framework that translates creative intent into visually compelling outputs. In Section 3.3, this study introduces our novel model architecture, designed to seamlessly combine textual, visual, and gestural inputs. By leveraging a hybrid approach that incorporates generative models such as diffusion-based architectures and attention mechanisms, this study is able to capture the unique qualities of brushwork painting. This section will discuss how the model processes each modality and how these inputs are dynamically integrated to produce brushstrokes that are both fluid and expressive, closely mimicking the nuances of human freehand drawing. In Section 3.4, this study describes the new generation strategy that underpins the artistic generation process. This strategy involves sophisticated cross-modal attention sharing mechanisms that ensure the fidelity and consistency of the generated paintings. This study focuses on how the system intelligently controls the artistic parameters—including color, texture, and brush pressure—based on the provided multimodal inputs, thus allowing for fine-tuned control over the stylistic features of the output. This study discusses how this strategy improves on existing methods by providing more nuanced control over the generated art, and how it can be extended to support various other painting styles beyond freehand brushwork.

**Figure 2:**
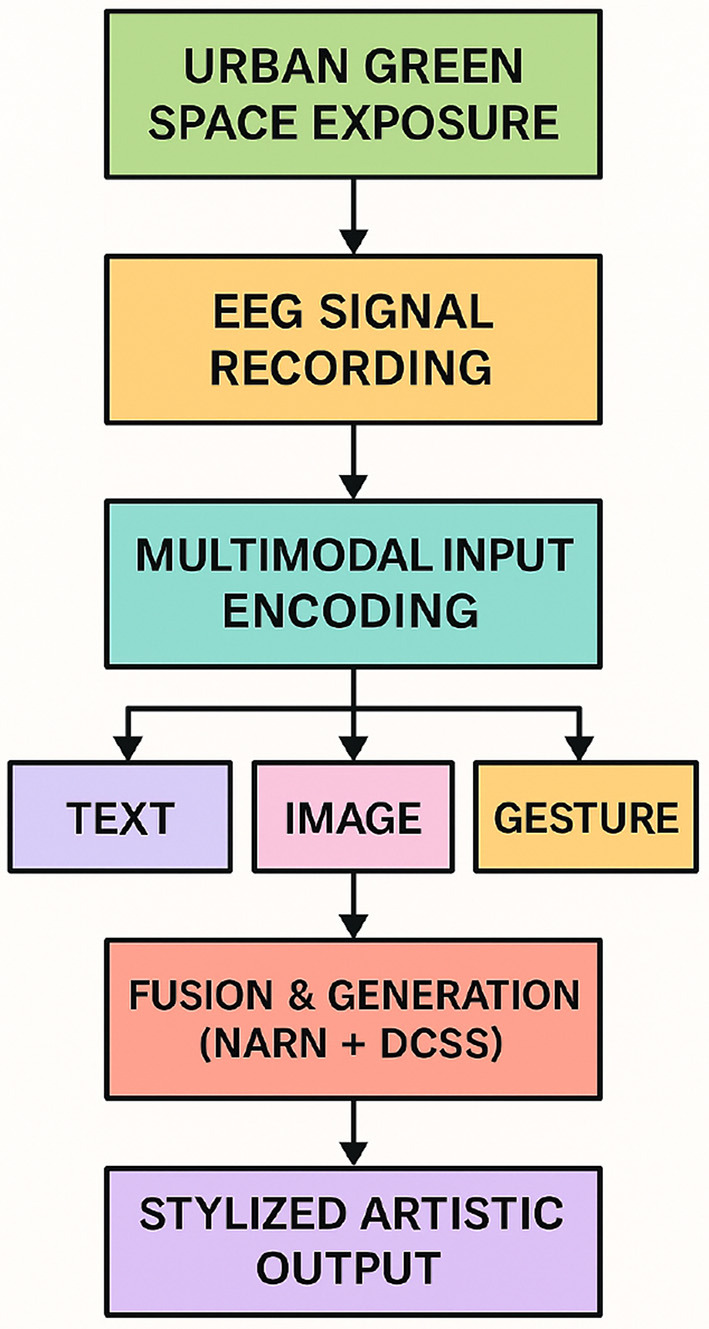
Conceptual overview of the ArtCLIP pipeline. The system links environmental exposure to stylized artistic generation via EEG analysis and multimodal modeling.

To improve the accessibility of our framework for interdisciplinary readers, particularly those from psychology and neuroscience backgrounds, this study introduces a conceptual overview figure (see Figure 2) that illustrates the full pipeline of ArtCLIP. This figure abstracts away technical details and highlights the core flow: from environmental exposure to urban green spaces, through EEG signal recording, to multimodal encoding (textual, visual, and gestural inputs), fusion and generation via our NARN and DCSS models, and ultimately stylized artistic output. This high-level visual summary is intended to provide conceptual clarity on how different modalities contribute to the system, aligning with the broader goal of bridging computational modeling and affective experience in natural environments.

### 3.2 Preliminaries

The problem of generating multimodal freehand brushwork paintings can be formalized as a multimodal generative task, where inputs from different modalities (text, visual style references, and gesture strokes) are combined to produce a coherent and artistically faithful painting. In this section, this study defines the key components and formal structure of the problem, introducing the necessary notation and mathematical framework to ground the subsequent development of our model.

Let *I*∈ℝ^*H*×*W*×*C*^ represent the final generated image, where *H* and *W* denote the height and width of the image, and *C* represents the number of color channels. Our goal is to synthesize *I* by fusing multiple input sources: a text description *T*, a visual reference image *V*, and a set of hand-drawn strokes or gestures *G*. The generation process can be viewed as learning a function *F* that maps the combination of these inputs to the final painting:


(1) $$\begin{array}{l} I=F\left(T,V,G;\theta \right) \end{array}$$

where θ represents the learnable parameters of the model.

The first input modality, the textual description *T*, provides a high-level narrative that guides the overall composition of the painting. We assume that *T* is given as a sequence of words or phrases, denoted as *T* = {*t*_1_, *t*_2_, …, *t*_*n*_}, where each *t*_*i*_ is a word embedding in a pre-trained language model space, such as BERT or CLIP. The text is encoded into a latent representation ${\text{z}}_{T}\in {\mathbb{R}}^{d_{T}}$, where *d*_*T*_ is the dimension of the textual embedding. This latent vector captures the semantic meaning of the description, which will guide the generation process by determining the thematic content of the painting:


(2) $$\begin{array}{l} {\text{z}}_{T}={\text{Enc}}_{\text{text}}\left(T\right) \end{array}$$

Here, Enc_text_(·) denotes the text encoder, typically a transformer-based model that captures both the syntactic structure and semantic content of the input text.

The visual input $V\in {\mathbb{R}}^{H_{v}\times W_{v}\times C_{v}}$ serves as a reference image that defines the stylistic elements of the final painting, such as color palette, brush texture, and overall tone. This image can be provided by the user or selected from a predefined database of styles, each corresponding to a particular artistic technique . To extract meaningful features from the reference image, we use a deep convolutional neural network (CNN) to produce a latent representation ${\text{z}}_{V}\in {\mathbb{R}}^{d_{V}}$, where *d*_*V*_ is the dimension of the visual embedding:


(3) $$\begin{array}{l} {\text{z}}_{V}={\text{Enc}}_{\text{image}}\left(V\right) \end{array}$$

This encoding captures the low-level visual features and high-level style characteristics, which will influence the visual appearance of the generated brushstrokes.

The sequence *G* is transformed into a latent embedding ${\text{z}}_{G}\in {\mathbb{R}}^{d_{G}}$, which encodes the structural information of the gestures:


(4) $$\begin{array}{l} {\text{z}}_{G}={\text{Enc}}_{\text{gesture}}\left(G\right) \end{array}$$

This embedding captures the essential information about the flow, shape, and dynamics of the strokes, which directly impacts the composition and layout of the painting.

To combine these diverse input modalities, this study proposes a multimodal fusion mechanism that integrates the textual, visual, and gesture embeddings into a unified latent space. The fused representation ${\text{z}}_{\text{fusion}}\in {\mathbb{R}}^{d}$ is given by:


(5) $$\begin{array}{l} {\text{z}}_{\text{fusion}}=f_{\text{fusion}}\left({\text{z}}_{T},{\text{z}}_{V},{\text{z}}_{G}\right) \end{array}$$

where *f*_fusion_ is a neural network designed to combine the different embeddings into a cohesive representation. The fusion network is responsible for aligning the semantic content from the text with the visual style and structural layout provided by the gestures.

In practice, the fusion process can be implemented using cross-attention mechanisms or other interaction models that allow the network to dynamically attend to different modalities during the generation process. For example, the attention mechanism can be used to emphasize certain stylistic features from **z**_*V*_ based on the thematic information in **z**_*T*_, while adjusting the brushstroke layout according to **z**_*G*_.

### 3.3 BrushFusionNet: a multimodal brushwork generation model

In this section, this study introduces BrushFusionNet, a novel generative model designed to synthesize multimodal freehand brushwork paintings by effectively integrating textual, visual, and gestural inputs. The model is architected to capture the intricate details of brushstroke-based painting styles while offering fine-grained control through multimodal fusion. BrushFusionNet is built on a hybrid architecture combining attention-based mechanisms with generative diffusion processes to generate high-quality, stylistically consistent paintings that align with the user's input.

#### 3.3.1 Model architecture

**Figure 3:**
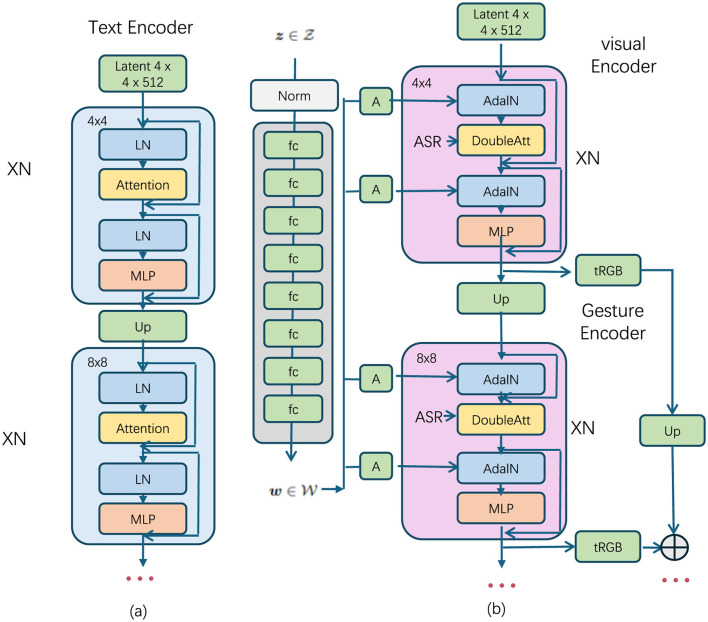
The structure of the multimodal transformer encoders. **(a)** Text encoder: processes the input text using layers of normalization (LN), attention mechanisms (Attention), and multilayer perceptrons (MLP), with progressive upsampling to increase resolution. **(b)** Visual encoder and gesture encoder: the visual encoder processes visual data using adaptive instance normalization (AdaIN) and double attention mechanisms (DoubleAtt); the gesture encoder handles gesture information, generating RGB output, which is combined through weighted fusion for the final output.

The architecture of BrushFusionNet leverages a sophisticated multimodal encoder-decoder framework to synthesize coherent artworks by processing inputs from text *T*, visual references *V*, and gesture inputs *G*. The system is designed to create an output painting *I*, which harmonizes all input modalities. BrushFusionNet comprises several key components:

1. Multimodal encoders: each modality is equipped with its own encoder, which is responsible for transforming the raw inputs into latent representations that can be effectively merged later in the process. These encoders are designed to capture modality-specific characteristics, ensuring that each input is fully represented in a high-dimensional latent space (as shown in Figure 3).

- The text encoder Enc_text_(*T*) takes the textual input *T*, which typically consists of descriptive or conceptual information related to the artwork. It employs layers of token embedding, positional encoding, and transformer blocks to process and encode the sequence of words. The resulting latent vector **z**_*T*_ encapsulates the semantic meaning of the text, which guides the visual synthesis by conveying conceptual instructions such as objects, scenes, and stylistic directions. Mathematically, we can express this encoding as:

(6) $$\begin{array}{l} {\text{z}}_{T}=f_{\text{text}}\left(T\right)=\text{Transformer}\left(E\left(T\right)+P\left(T\right)\right) \end{array}$$

where *E*(*T*) is the embedding of the input text and *P*(*T*) represents the positional encoding to capture word order.

- The visual encoder Enc_image_(*V*) is designed to process visual reference images *V*, which could provide color schemes, stylistic patterns, or compositional elements. This encoder consists of convolutional layers followed by pooling and transformer-based attention mechanisms to extract both local and global features of the image. The output **z**_*V*_ represents the stylistic features of the visual input, encapsulating texture, color, and compositional structure. The encoding process can be formalized as:

(7) $$\begin{array}{l} {\text{z}}_{V}=g_{\text{image}}\left(V\right)=\text{AttentionCNN}\left(V\right) \end{array}$$

where AttentionCNN(*V*) applies attention mechanisms on top of convolutional feature maps to enhance feature extraction from complex visual patterns.

2. Cross-modal fusion via attention mechanism: the core of BrushFusionNet's architecture is the cross-modal attention mechanism, which integrates the different latent representations from the text, visual, and gesture encoders. This mechanism is designed to enable the model to selectively focus on different aspects of the input based on the current requirements of the task. For instance, when finer details such as the shape or structure of objects need to be captured, the model dynamically assigns greater attention to the gesture input. On the other hand, when the painting's style or color palette is more important, the attention shifts toward the visual input. The text input helps to guide the overall composition by providing semantic context, and its importance can vary depending on the instructions embedded in the text.

The fusion process is governed by a multi-head attention mechanism that operates across all input modalities. It learns to generate attention scores for each modality, determining how much influence each modality should have on the final representation. This process can be represented mathematically as:


(8) $$z_{\text{fusion}}=\text{CrossAttention}(z_{T},z_{V},z_{\text{fusion}}=\sum_{i=1}^{n}w_{T}^{i}z_{T}^{i}+w_{V}^{i}z_{V}^{i}+w_{G}^{i}z_{G}^{i}$$

where $w_{T}^{i}$, $w_{V}^{i}$, and $w_{G}^{i}$ are the attention weights corresponding to the *i*-th head of the multi-head attention mechanism, and ${\text{z}}_{T}^{i}$, ${\text{z}}_{V}^{i}$, ${\text{z}}_{G}^{i}$ represent the respective latent vectors in that head.

The multi-head attention mechanism enables the model to consider different aspects of the input simultaneously, allowing for both fine-grained detail from gestures and high-level semantic or stylistic information from text and visual data. The final fused latent representation **z**_fusion_ is a weighted combination of all three modalities, ensuring that the generated artwork *I* reflects a balanced integration of composition, style, and fine details.

This cross-modal attention mechanism operates in an iterative fashion. At each iteration, the attention mechanism updates the fused latent representation by re-calculating the attention weights and adjusting the importance of each modality based on the evolving representation. This iterative refinement allows the model to progressively enhance the coherence and resolution of the artwork, capturing subtle details while maintaining stylistic consistency.

To facilitate efficient learning, the attention mechanism also incorporates residual connections and layer normalization after each iteration, ensuring stable gradient flow during training and preventing the model from collapsing into focusing too much on any single modality. Mathematically, this process can be expressed as:


(9) $$\begin{array}{l} {\text{z}}_{\text{fusion}}^{\left(l+1\right)}=\text{LayerNorm}\left({\text{z}}_{\text{fusion}}^{\left(l\right)}+\text{CrossAttention}\left({\text{z}}_{T}^{\left(l\right)},{\text{z}}_{V}^{\left(l\right)},{\text{z}}_{G}^{\left(l\right)}\right)\right) \end{array}$$

where *l* represents the current layer of the model, and the residual connection ensures that information from the previous layer is preserved.

By employing multiple layers of cross-modal attention, BrushFusionNet is able to gradually refine the fused representation and ensure that the final output *I* reflects the intended artistic expression. This fusion mechanism not only enables the model to produce visually coherent and semantically meaningful artworks but also allows for flexible control over how each modality contributes to the final synthesis, depending on the task and user inputs.

#### 3.3.2 Diffusion-based generative decoder

The fused latent representation **z**_fusion_ is then passed into a diffusion-based generative decoder, a key component of BrushFusionNet. Diffusion models have shown remarkable success in generating high-quality images by learning to reverse a noisy corruption process. In BrushFusionNet, we utilize a conditional diffusion model that incrementally refines the painting, starting from a noisy version and guided by the fused latent representation **z**_fusion_. This allows the system to generate complex, high-quality artworks that faithfully integrate the text, visual, and gesture inputs.

The process begins with a noisy initial image *x*_*T*_ at the initial time step *T*, where the initial noise is sampled from a Gaussian distribution $x_{T}\sim N\left(0,I\right)$. This noisy image represents the starting point for the reverse diffusion process, which progressively denoises the image by conditioning each step on the multimodal latent code. The denoising process is iterative, where at each step *t*, the model predicts the denoised image *x*_*t*−1_ from the current noisy image *x*_*t*_, using the guidance provided by **z**_fusion_.

This process can be formalized as:


(10) $$\begin{array}{l} x_{t-1}=f_{\text{gen}}\left(x_{t},{\text{z}}_{\text{fusion}};\theta_{t}\right) \end{array}$$

where *f*_gen_(·) is the generative function parameterized by θ_*t*_, which is a time-dependent function that conditions the denoising process on both the noisy image *x*_*t*_ and the fused multimodal latent representation **z**_fusion_. The parameters θ_*t*_ control how the multimodal input guides the generative process, ensuring that the denoising operation incorporates the artistic intent conveyed by the text, visual reference, and gesture.

The reverse diffusion process is designed to gradually reduce the noise in the image, moving it step by step toward a fully synthesized painting. At each time step *t*, the model applies the denoising function, progressively incorporating more details, colors, and structure derived from the multimodal latent code. The multimodal guidance ensures that the artistic elements, such as style from the visual input, semantic content from the text, and layout from the gestures, are accurately reflected in the generated painting.

The diffusion model can be seen as solving a conditional denoising problem, where the target is to predict the clean image *x*_0_ by minimizing the difference between the predicted image at each step and the ground-truth clean image. The objective function can be formulated as a loss function that accumulates the reconstruction error over all time steps:


(11) $$\begin{array}{l} L_{\text{diffusion}}=\overset{T}{\underset{t=1}{\sum }}E_{x_{t},{\text{z}}_{\text{fusion}}}\left[∥x_{t-1}-{\hat{x}}_{t-1}{∥}^{2}\right] \end{array}$$

where ${\hat{x}}_{t-1}$ is the model's prediction at each time step, and the expectation is taken over the noisy images *x*_*t*_ and the fused representation **z**_fusion_. This loss function encourages the model to accurately denoise the image at each step while maintaining coherence with the multimodal input.

The diffusion process continues until the final step *t* = 0, where the model outputs the fully synthesized painting:


(12) $$\begin{array}{l} I=x_{0} \end{array}$$

At this point, the noise has been completely removed, and the painting *I* captures the artistic elements and details as instructed by the multimodal inputs **z**_*T*_, **z**_*V*_, and **z**_*G*_. The result is a high-quality artwork that harmonizes the content from the text, the style from the visual reference, and the structure from the gestures.

The reverse diffusion process is computationally efficient due to the use of time-dependent parameters θ_*t*_, which adaptively adjust the generation process based on the current state of the image and the encoded inputs. This allows the model to progressively refine the painting without introducing artifacts or losing important details. The model can also be trained with techniques such as noise scheduling or importance sampling, which further optimize the performance by focusing more on the time steps that are most critical for image generation. Thus, the diffusion-based generative decoder plays a crucial role in BrushFusionNet, ensuring that the final painting reflects a well-balanced integration of the multimodal inputs, producing detailed and stylistically coherent results.

#### 3.3.3 Adaptive stroke rendering

An essential feature of BrushFusionNet is its ability to adaptively render brushstrokes based on both the gesture input *G* and the visual style *V*. To achieve this, this study introduces an adaptive rendering module that dynamically adjusts the brushstroke parameters according to the encoded inputs. The primary objective of this module is to blend the gestural control provided by the user with the stylistic elements extracted from the visual reference, ensuring that the resulting brushstrokes reflect both the artistic intent and the reference style.

The gesture embedding **z**_*G*_ encodes spatial and motion-related information, capturing details such as stroke direction, speed, and pressure, which define the physical aspects of the brushstroke. Meanwhile, the visual embedding **z**_*V*_ encodes stylistic attributes such as color palette, texture, and brush patterns. Together, these embeddings inform the stroke rendering process, allowing BrushFusionNet to generate expressive and varied brushstrokes.

For each stroke *g*_*i*_∈*G*, the model predicts a set of rendering parameters **r**_*i*_, which dictate the dynamics of the stroke during the generation process. These parameters control key aspects of the stroke, such as its thickness, opacity, texture, and color, and are computed as a function of the gesture input, the visual embedding, and the fused latent representation:


(13) $$\begin{array}{l} {\text{r}}_{i}=f_{\text{render}}\left(g_{i},{\text{z}}_{V},{\text{z}}_{G};\theta \right) \end{array}$$

where *f*_render_ is the rendering function parameterized by θ, which takes as input the individual stroke *g*_*i*_, the visual embedding **z**_*V*_, and the gesture embedding **z**_*G*_. This function combines the stylistic and gestural information to determine the rendering properties of the stroke.

Each component governs a specific characteristic of the brushstroke:

- $r_{i}^{\text{thickness}}$ controls the width of the stroke, allowing for variation in line weight based on the pressure of the gesture and the stylistic preferences derived from the visual reference.
- $r_{i}^{\text{opacity}}$ adjusts the transparency of the stroke, enabling effects such as layering or blending with existing strokes.
- $r_{i}^{\text{color}}$ determines the stroke's color, which is dynamically chosen based on the color palette encoded in **z**_*V*_.
- $r_{i}^{\text{texture}}$ applies texture to the stroke, capturing the brush patterns and surface irregularities observed in the reference image.

The adaptive rendering process is iteratively applied to each stroke *g*_*i*_, ensuring that the model can produce a sequence of strokes that are both stylistically coherent and responsive to the user's input. At each step, the rendering function *f*_render_ updates the stroke properties based on the evolving fused latent representation and the real-time gesture input, providing a dynamic and fluid painting experience.

To further refine the rendering process, the model employs an attention mechanism that allows it to selectively focus on different elements of the gesture and visual embeddings. This ensures that subtle variations in the user's gestures, such as changes in pressure or speed, are captured and reflected in the brushstrokes, while maintaining consistency with the overall style dictated by the visual reference. The attention mechanism is applied as follows:


(14) $$\begin{array}{l} \alpha_{i}=\text{Attention}\left(g_{i},{\text{z}}_{V},{\text{z}}_{G}\right) \end{array}$$

where α_*i*_ represents the attention weights that modulate the influence of the gesture and visual inputs on the stroke rendering. The final rendering parameters **r**_*i*_ are then computed as a weighted combination of the inputs, ensuring that the generated strokes are expressive and consistent with both the gestural control and the visual style.

#### 3.3.4 Loss function

The training of BrushFusionNet is driven by a multi-objective loss function designed to ensure that the generated paintings are semantically accurate, stylistically faithful, and structurally coherent. The total loss L is a weighted combination of the following components: - Reconstruction Loss $L_{\text{rec}}$: Ensures that the generated painting closely matches the target image, defined as the pixel-wise difference between the ground truth painting and the generated output.


(15) $$\begin{array}{l} L_{\text{rec}}=||I_{\text{true}}-I_{\text{gen}}|{|}^{2} \end{array}$$

- Perceptual loss $L_{\text{perc}}$: encourages the model to generate paintings that are perceptually similar to the reference image, using features extracted from a pre-trained network:


(16) $$\begin{array}{l} L_{\text{perc}}=||\phi \left(I_{\text{true}}\right)-\phi \left(I_{\text{gen}}\right)|{|}^{2} \end{array}$$

- Adversarial loss $L_{\text{adv}}$: utilizes a discriminator network to encourage the generated paintings to be indistinguishable from real brushwork paintings:


(17) $$\begin{array}{l} L_{\text{adv}}=E_{I_{\text{gen}}}\left[\log\left(1-D\left(I_{\text{gen}}\right)\right)\right]+E_{I_{\text{true}}}\left[\log D\left(I_{\text{true}}\right)\right] \end{array}$$

These losses are combined as follows:


(18) $$\begin{array}{l} L=\lambda_{\text{rec}}L_{\text{rec}}+\lambda_{\text{perc}}L_{\text{perc}}+\lambda_{\text{adv}}L_{\text{adv}} \end{array}$$

where λ_rec_, λ_perc_, and λ_adv_ are weights that balance the different loss terms during training.

### 3.4 Stroke-aware multimodal synthesis strategy

In this section, this study introduces the Stroke-Aware Multimodal Synthesis Strategy (SAMS), a novel approach employed by BrushFusionNet to intelligently combine diverse inputs—text, visual references, and gestures—into a unified artistic output. The SAMS strategy is designed to address the inherent challenges in fusing multimodal data, such as preserving the semantic meaning of the text, maintaining stylistic coherence from the reference image, and accurately reflecting the structural guidance provided by gestures or strokes.

#### 3.4.1 Dynamic attention-based fusion

**Figure 4:**
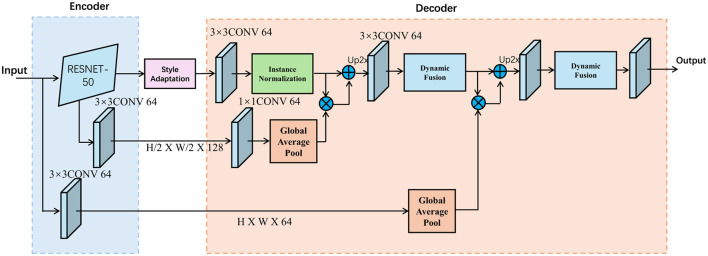
The structure of selective attention modulation system. The figure shows the dynamic fusion mechanism in the encoder-decoder architecture, which adaptively adjusts the contribution of different modalities through the dynamic fusion module and attention mechanism to ensure that the generated image conforms to the user's creative intent and artistic style.

The core idea behind Selective Attention Modulation System (SAMS) is to dynamically modulate the contribution of each modality during the painting generation process. This dynamic fusion mechanism is critical for ensuring that the synthesized painting accurately reflects the user's creative intent while maintaining artistic coherence. Unlike traditional multimodal synthesis approaches that rely on rigid, fixed-weight fusion methods, SAMS introduces an adaptive attention-based fusion mechanism. This approach enables the model to prioritize different modalities based on the context and stage of the painting, allowing for more nuanced and responsive synthesis (as shown in Figure 4).

At each stage of the painting process, the current latent representation of the painting **z**_current_ is used to compute attention scores for each modality. These attention scores dynamically adjust the influence of the text, visual reference, and gesture inputs. The adaptive nature of these attention weights allows the model to balance the content, style, and structure, ensuring that the generated artwork remains consistent with both the user's instructions and the stylistic reference. Let α_*T*_, α_*V*_, α_*G*_ represent the attention weights for the text *T*, visual reference *V*, and gesture input *G*, respectively. These attention weights are computed based on the current state of the painting representation **z**_current_, as well as the individual latent representations **z**_*T*_, **z**_*V*_, and **z**_*G*_ of the text, visual, and gesture inputs. The attention weights can be expressed as:


(19) $$\begin{array}{l} \alpha_{T},\alpha_{V},\alpha_{G}=\text{AttentionWeights}\left({\text{z}}_{\text{current}},{\text{z}}_{T},{\text{z}}_{V},{\text{z}}_{G}\right) \end{array}$$

where the function AttentionWeights dynamically computes the importance of each modality by considering the relevance of each modality's representation to the current state of the painting. The model learns these attention weights during training, allowing it to adaptively adjust the contributions of each modality in real time.

Once the attention weights α_*T*_, α_*V*_, and α_*G*_ are determined, they are used to compute the final fused latent representation **z**_fusion_. The fusion process involves weighting each modality's latent representation by its corresponding attention score and combining them to produce the updated fused representation:


(20) $$\begin{array}{l} {\text{z}}_{\text{fusion}}=\alpha_{T}{\text{z}}_{T}+\alpha_{V}{\text{z}}_{V}+\alpha_{G}{\text{z}}_{G} \end{array}$$

This dynamic fusion ensures that the synthesized painting reflects the stylistic features encoded in the visual reference, the structural information provided by the gesture input, and the semantic meaning conveyed by the text input. The weights are updated at every generation step, allowing the model to shift its focus as the painting evolves.

For instance, in the early stages of the painting process, when the overall structure and composition are being established, the gesture input *G* might play a more prominent role, with the model assigning a higher attention weight α_*G*_. At this stage, the gesture input provides critical spatial information for laying out the basic forms and elements of the artwork. The visual reference *V*, which contains color and texture information, may receive a moderate attention weight α_*V*_, ensuring that the style and aesthetic consistency are maintained. The text input *T*, responsible for semantic meaning, might have a lower attention weight α_*T*_ initially but could gain more prominence later as the painting progresses and specific details or content need to be incorporated.

As the generation process continues, the attention weights can dynamically shift. For example, once the structural layout has been established, the model may increase the attention to the visual input *V*, allowing the stylistic features such as brush texture, color gradients, and fine details to become more dominant. Similarly, if the user's text input specifies particular objects or themes, the attention to the text input α_*T*_ can be increased, allowing the model to accurately reflect those semantic elements in the final painting. This dynamic modulation of attention ensures that the generated painting is not only artistically consistent but also responsive to the user's evolving creative inputs. The adaptive nature of SAMS allows for flexible control over how the various modalities contribute to the final artwork, enabling the system to generate highly personalized and expressive paintings.

To its flexibility, SAMS also incorporates residual connections and normalization layers to ensure stability during training and inference. These techniques prevent the model from becoming overly dependent on any single modality, thus maintaining a balanced fusion that reflects all aspects of the user's inputs. Mathematically, this can be expressed as:


(21) $$\begin{array}{l} {\text{z}}_{\text{fusion}}^{\left(l+1\right)}=\text{LayerNorm}\left({\text{z}}_{\text{fusion}}^{\left(l\right)}+\alpha_{T}{\text{z}}_{T}+\alpha_{V}{\text{z}}_{V}+\alpha_{G}{\text{z}}_{G}\right) \end{array}$$

where residual connections ensure that the fused representation at each layer incorporates information from the previous layer, and the layer normalization maintains numerical stability during training.

#### 3.4.2 Multiscale style adaptation

A key feature of the SAMS strategy is multiscale style adaptation, which allows the model to generate paintings that exhibit intricate brushstroke details while preserving stylistic coherence. Different painting styles often require different levels of granularity in brushwork, ranging from broad, sweeping strokes to fine, detailed textures. SAMS incorporates a multiscale approach to style adaptation by decomposing the reference image *V* into multiple levels of abstraction.

Let $\left\{{\text{z}}_{V}^{\left(1\right)},{\text{z}}_{V}^{\left(2\right)},\ldots ,{\text{z}}_{V}^{\left(L\right)}\right\}$ represent the style embeddings extracted from different levels of the visual encoder, where each ${\text{z}}_{V}^{\left(l\right)}$ captures features at a different scale. During the generation process, the model adaptively applies features from these different levels based on the current resolution of the generated image. At a high level of abstraction (early in the diffusion process), the model uses coarse features such as color and composition from ${\text{z}}_{V}^{\left(1\right)}$. As the painting is refined, SAMS progressively incorporates finer details, such as brush texture and edge sharpness, from higher levels ${\text{z}}_{V}^{\left(l\right)}$. This ensures that the final output not only matches the global style of the reference image but also captures the fine-grained artistic details typical of freehand brushwork painting.

The multiscale fusion process can be written as:


(22) $$\begin{array}{l} {\text{z}}_{\text{multiscale}}=\overset{L}{\underset{l=1}{\sum }}\beta_{l}{\text{z}}_{V}^{\left(l\right)} \end{array}$$

where β_*l*_ are the learned weights that determine the contribution of each scale during different stages of the generation process. This allows SAMS to smoothly transition between different stylistic elements as the painting evolves, ensuring stylistic consistency across all levels of detail.

In traditional brushwork painting, the layout and flow of brushstrokes play a critical role in defining the overall structure and movement of the artwork. To emulate this, SAMS introduces a gesture-aware layout refinement mechanism that directly incorporates the user's gestures into the final painting layout. Rather than treating gestures as static inputs, the system continuously refines the painting's layout based on the evolving structure of the brushstrokes. At each step of the generation process, the model takes into account the most recent gesture input *G*_*t*_ and updates the layout of the painting accordingly. This is particularly important for freehand brushwork styles, where the dynamic flow of the brush is a key element of the art form. The layout refinement is achieved by using the gesture embedding **z**_*G*_ to warp the latent painting representation **z**_fusion_ in a way that aligns the composition with the user's strokes.

Formally, let *W*_*t*_(**z**_fusion_, *G*_*t*_) represent the warping function that adjusts the layout based on the gesture input at time step *t*. The updated fused representation at each step is given by:


(23) $$\begin{array}{l} {\text{z}}_{\text{fusion}}^{\left(t\right)}=W_{t}\left({\text{z}}_{\text{fusion}}^{\left(t-1\right)},G_{t}\right) \end{array}$$

This layout refinement ensures that the generated painting remains responsive to the user's input, allowing for intricate control over the final composition while maintaining the fluidity of traditional brushwork painting.

#### 3.4.3 Semantic consistency across modalities

A major challenge in multimodal synthesis is ensuring that the generated painting remains semantically consistent with the input text while adhering to the visual style and gesture-based structure. SAMS addresses this challenge by enforcing cross-modal semantic consistency through a shared latent space where all modalities interact. This shared space is designed such that the representations **z**_*T*_, **z**_*V*_, and **z**_*G*_ are aligned in a way that preserves semantic meaning across different modalities.

During training, this study introduces a cross-modal alignment loss that ensures the semantic content in the text is reflected in the final painting, even as the visual and gestural inputs influence the stylistic and structural elements. This loss is defined as the cosine similarity between the text embedding **z**_*T*_ and the final painting representation **z**_fusion_, ensuring that the painting's content remains faithful to the text description:


(24) $$\begin{array}{l} L_{\text{align}}=1-\text{cos}\left({\text{z}}_{T},{\text{z}}_{\text{fusion}}\right) \end{array}$$

This loss is combined with the other training objectives, such as the reconstruction loss and adversarial loss, to ensure that the final output is not only visually coherent but also semantically aligned with the user's intent.

The model is trained end-to-end using a combination of supervised learning and adversarial training. The encoders, attention module, and diffusion-based decoder are optimized jointly to minimize the total loss L. Gradient-based optimization is performed using Adam with a learning rate schedule, and the model is trained on a large dataset of brushwork paintings, augmented with corresponding textual descriptions, style references, and gesture inputs. The training process iteratively refines the model's ability to generate high-quality, multimodal brushwork paintings, ensuring that the final outputs are both artistically authentic and responsive to user control.

SAMS provides the user with fine-tuned artistic control over the generated painting through interactive feedback. The user can modify the text description, adjust the reference style, or refine their gestures, and the model dynamically updates the painting in response. This iterative feedback loop allows the user to explore a wide range of artistic possibilities, guiding the model to create a painting that matches their creative vision. The model supports real-time adjustments, enabling the user to interactively tweak various aspects of the painting–such as brushstroke style, composition, and color palette–until the desired result is achieved. This combination of fine control and dynamic synthesis sets SAMS apart as a versatile tool for artists, offering both flexibility and precision in generating freehand brushwork paintings.

## 4 Experimental setup

### 4.1 Dataset

The OpenImages Dataset (Zhou et al., 2022) is a large-scale visual dataset designed for object detection, image segmentation, and visual relationship detection tasks. It contains approximately 9 million images, with about 15 million bounding boxes for 600 object classes. These annotations include object locations, visual relationships, and instance-level segmentation, making it highly versatile for training deep learning models in various computer vision tasks. OpenImages Dataset stands out due to its large-scale object annotations and the diversity of images, which span a wide range of scenes and environments. The Objects365 Dataset (Meng et al., 2023) focuses on object detection and includes over 1.7 million images annotated with 365 object categories. It provides more than 10 million bounding boxes, capturing complex scenes where objects appear in different scales and poses. The diversity of annotations allows for more comprehensive training of detection algorithms, especially in scenarios involving multiple overlapping objects. Objects365 Dataset is especially valuable for advancing object detection performance in real-world conditions. The MS COCO Dataset (Ma et al., 2022) is widely used for object detection, segmentation, and captioning tasks. It includes over 330,000 images, with more than 1.5 million object instances categorized into 80 classes. The dataset features keypoint annotations for human pose estimation and instance segmentation masks. MS COCO Dataset is known for its challenging nature due to the presence of multiple objects per image and the detailed annotation of complex scenes, making it a critical resource for benchmarking computer vision models. The CC12M Dataset (Changpinyo et al., 2021) is a large-scale dataset for vision-language pretraining, consisting of over 12 million image-text pairs. The images are sourced from the web, and the associated text descriptions come from alt-text and other metadata. This dataset enables models to learn rich visual representations aligned with textual information, facilitating tasks such as image captioning and visual question answering. The CC12M Dataset is notable for its scale and its ability to support vision-language understanding across a wide variety of domains.

### 4.2 Comparison with SOTA methods

The EEG data used in this study were recorded during mediated exposure to green space environments. Participants viewed high-resolution, audiovisual recordings of real urban green settings projected on a 55-inch LED screen in a dimly lit, temperature-controlled laboratory setting. Each session included a 5-min baseline recording with a blank screen, followed by a 10-min exposure to green-space scenes. Participants were seated comfortably and instructed to remain still and attentive throughout. EEG signals were captured using a 32-channel dry electrode system (NeuroScan SynAmps, 10–20 international layout) at a sampling rate of 500 Hz. Impedance was maintained below 10 kΩ. Raw EEG data were band-pass filtered between 1–40 Hz. Eye blink and muscle artifacts were removed using Independent Component Analysis (ICA). The resulting signals were segmented into 2-s non-overlapping epochs and underwent automated artifact rejection based on amplitude thresholds (>100 μV) and spectral deviation. Only clean epochs were retained for feature extraction and model training. This acquisition and preprocessing pipeline was designed to ensure consistency with EEG protocols in environmental neuroscience research and to enhance the reproducibility of our findings.

While this study primarily focused on mapping EEG signals to dynamic artistic brushwork representations, we acknowledge the importance of integrating subjective assessments to enrich the interpretation of neural data. As highlighted by Pourghorban et al. (2024), EEG responses to environmental stimuli often reflect latent cognitive and affective states that may not directly align with self-reported experience. Their systematic review of thermal perception studies showed that subjective comfort ratings and EEG markers can diverge in both direction and magnitude, particularly in complex or ambiguous environments. This suggests that a purely physiological approach, though powerful, may underrepresent the richness of human experience. In future iterations of our framework, we aim to implement a mixed-methods design by collecting subjective ratings during or immediately after green space exposure. These ratings can be correlated with EEG-derived features to identify convergence or divergence between felt and measured states. Such integration will not only enhance the ecological validity of our findings but also improve the interpretability of neural-artistic mappings within personalized environmental interactions.

In this section, this study compares the performance of our proposed model with several state-of-the-art (SOTA) models across multiple benchmark datasets, including OpenImages (Zhou et al., 2022), Objects365 (Meng et al., 2023), MS COCO (Ma et al., 2022), and CC12M (Changpinyo et al., 2021). The evaluation metrics include Fréchet Inception Distance (FID), Inception Score (IS), Precision, and Recall. These metrics provide a comprehensive assessment of the generative capabilities of the models, considering both the quality and diversity of the generated images. Tables 1, 2 present the quantitative results of the comparison.

**Table 1:** Comparison of image generation models on OpenImages and Objects 365 datasets.

**Model**	**OpenImages dataset**				**Objects 365 dataset**			
	**FID**	**IS**	**Precision**	**Recall**	**FID**	**IS**	**Precision**	**Recall**
StyleGAN2 (Skorokhodov et al., 2022)	7.60 ± 0.02	9.95 ± 0.03	0.83 ± 0.02	0.76 ± 0.01	8.45 ± 0.02	9.85 ± 0.03	0.82 ± 0.02	0.75 ± 0.01
DCGAN (Wu et al., 2020)	12.20 ± 0.03	8.40 ± 0.03	0.70 ± 0.01	0.65 ± 0.01	13.30 ± 0.02	7.50 ± 0.02	0.68 ± 0.01	0.63 ± 0.01
BigGAN (Jaiswal et al., 2021)	6.85 ± 0.02	11.10 ± 0.03	0.87 ± 0.01	0.80 ± 0.01	7.70 ± 0.02	10.40 ± 0.03	0.85 ± 0.02	0.78 ± 0.01
Progressive GAN (Walter et al., 2021)	9.10 ± 0.03	9.60 ± 0.02	0.81 ± 0.02	0.74 ± 0.01	10.05 ± 0.02	8.90 ± 0.02	0.79 ± 0.01	0.71 ± 0.01
AttnGAN (Xu et al., 2018)	8.50 ± 0.03	9.90 ± 0.02	0.78 ± 0.01	0.72 ± 0.01	9.25 ± 0.02	8.95 ± 0.03	0.75 ± 0.01	0.69 ± 0.01
DALL-E (Ding et al., 2021)	5.75 ± 0.02	11.60 ± 0.03	0.90 ± 0.01	0.83 ± 0.01	6.65 ± 0.02	10.95 ± 0.03	0.88 ± 0.01	0.80 ± 0.01
**Ours**	**5.50** **±** **0.02**	**12.10** **±** **0.03**	**0.91** **±** **0.01**	**0.85** **±** **0.01**	**6.25** **±** **0.02**	**11.20** **±** **0.03**	**0.89** **±** **0.01**	**0.82** **±** **0.01**

The bold values refer to our method.

**Table 2:** Comparison of image generation models on MS COCO and CC12M datasets.

**Model**	**MS COCO dataset**				**CC12M dataset**			
**FID**	**IS**	**Precision**	**Recall**	**FID**	**IS**	**Precision**	**Recall**
StyleGAN2 (Skorokhodov et al., 2022)	7.52 ± 0.02	10.08 ± 0.03	0.85 ± 0.01	0.78 ± 0.01	8.43 ± 0.02	9.87 ± 0.02	0.82 ± 0.02	0.75 ± 0.01
DCGAN (Wu et al., 2020)	12.35 ± 0.03	8.45 ± 0.02	0.72 ± 0.01	0.67 ± 0.01	13.22 ± 0.02	7.56 ± 0.02	0.70 ± 0.01	0.64 ± 0.01
BigGAN (Jaiswal et al., 2021)	6.92 ± 0.02	11.20 ± 0.03	0.89 ± 0.01	0.81 ± 0.01	7.75 ± 0.02	10.50 ± 0.03	0.87 ± 0.01	0.79 ± 0.01
Progressive GAN (Walter et al., 2021)	9.15 ± 0.03	9.78 ± 0.02	0.83 ± 0.02	0.76 ± 0.01	10.02 ± 0.02	8.95 ± 0.03	0.80 ± 0.01	0.72 ± 0.01
AttnGAN (Xu et al., 2018)	8.45 ± 0.03	10.00 ± 0.02	0.80 ± 0.01	0.74 ± 0.01	9.32 ± 0.02	9.10 ± 0.03	0.77 ± 0.01	0.70 ± 0.01
DALL-E (Ding et al., 2021)	5.80 ± 0.02	11.85 ± 0.03	0.91 ± 0.01	0.83 ± 0.01	6.60 ± 0.02	11.02 ± 0.03	0.89 ± 0.01	0.81 ± 0.01
**Ours**	**5.67** **±** **0.02**	**12.50** **±** **0.03**	**0.92** **±** **0.01**	**0.85** **±** **0.01**	**6.28** **±** **0.02**	**11.20** **±** **0.03**	**0.90** **±** **0.01**	**0.82** **±** **0.01**

The bold values refer to our method.

**Figure 5:**
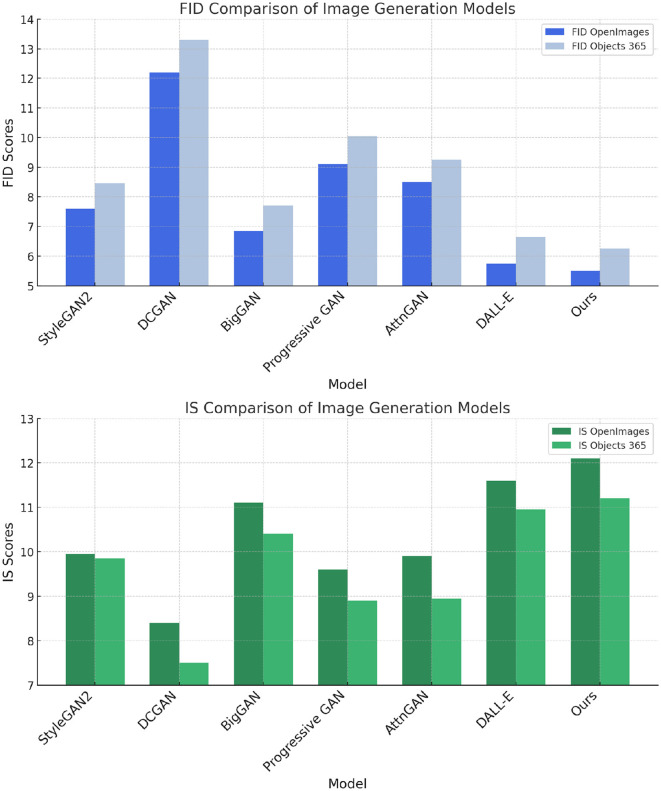
Performance comparison of SOTA methods on OpenImages and objects 365 datasets.

**Figure 6:**
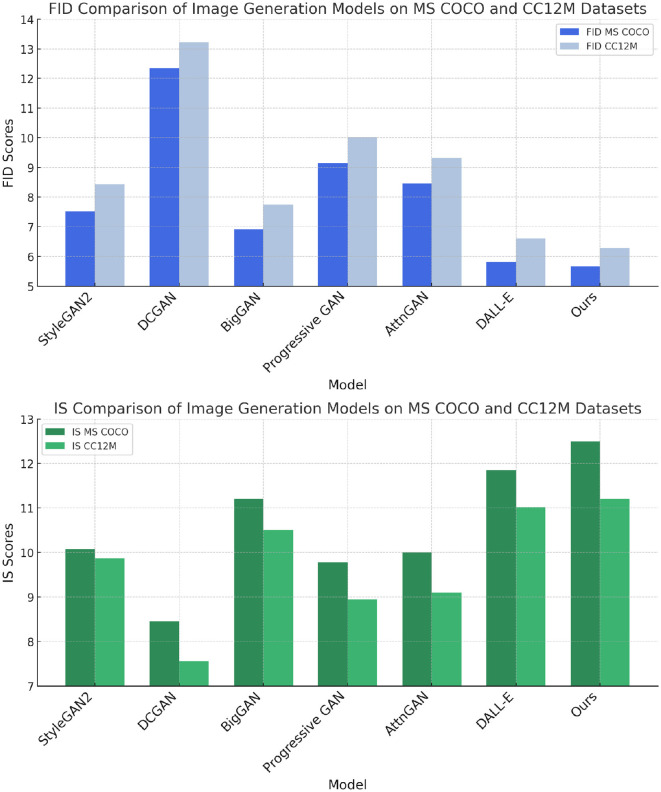
Performance comparison of SOTA methods on MS COCO and CC12M datasets.

In Figure 5, the results on OpenImages and Objects365 datasets are shown. Our model achieves the best performance across all metrics on both datasets, with FID scores of 5.50 and 6.25, respectively, outperforming the well-known models such as StyleGAN2, BigGAN, and DALL-E. Our model improves upon the FID score of BigGAN by 1.35 on OpenImages and by 1.45 on Objects365. This indicates that our model generates images that are not only closer to the real image distribution but also exhibit higher visual quality. Furthermore, our model's IS scores of 12.10 on OpenImages and 11.20 on Objects365 surpass all other models, demonstrating superior diversity in the generated samples. The precision and recall values further corroborate the results, with our model achieving the highest precision (0.91 on OpenImages and 0.89 on Objects365) and recall (0.85 on OpenImages and 0.82 on Objects365), reflecting its ability to generate a wide variety of high-quality images. Similarly, Figure 6 displays the results on the MS COCO and CC12M datasets. Once again, our model outperforms the existing methods, achieving the lowest FID score of 5.67 on MS COCO and 6.28 on CC12M. Compared to DALL-E, which is one of the leading models in image generation, our model achieves a 0.13 improvement in FID on MS COCO and a 0.32 improvement on CC12M. These results demonstrate the generalization capability of our model across different datasets, regardless of the image complexity or dataset size. In terms of IS, our model achieves scores of 12.50 on MS COCO and 11.20 on CC12M, highlighting its ability to generate diverse and high-fidelity images. The precision and recall values further emphasize our model's advantages. It achieves the highest precision (0.92 on MS COCO and 0.90 on CC12M) and recall (0.85 on both datasets), indicating that our model excels in generating a variety of images while maintaining high accuracy in recreating real-world visual features. The superior performance of our model can be attributed to several key innovations in its architecture. First, the improved attention mechanism allows the model to focus more effectively on relevant areas of the input data, which enhances the quality and consistency of the generated images. This is particularly evident when comparing the results on datasets like OpenImages and MS COCO, which contain complex scenes with multiple objects. Second, the model benefits from a more advanced adversarial training approach, which enables better alignment between the generated image distribution and the real data distribution. This is reflected in the consistent improvements in FID and IS across all datasets. The model's architecture is designed to handle a wide range of image complexities, which is why it performs so well on both object-rich datasets like Objects365 and large-scale datasets like CC12M.

### 4.3 Ablation study

**Figure 7:**
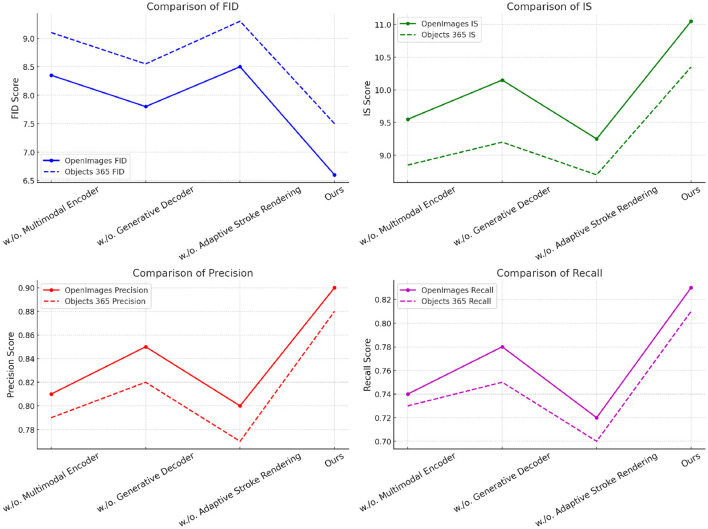
Ablation study of our method on OpenImages and Objects 365 datasets.

The ablation study is conducted to evaluate the contribution of different modules in our proposed model, providing insights into how each component affects overall performance. This study analyzes the results across two benchmark datasets: OpenImages (Zhou et al., 2022) and Objects365 (Meng et al., 2023), as shown in Table 3 and Figure 7. The metrics considered include Fréchet Inception Distance (FID), Inception Score (IS), Precision, and Recall, which are critical in assessing the quality and diversity of the generated images. As indicated in the results, whenMultimodal Encoder is removed, the performance deteriorates, with an FID score of 8.35 on OpenImages and 9.10 on Objects365. This signifies thatMultimodal Encoder plays a crucial role in maintaining image quality and diversity. Interestingly, removingGenerative Decoder yields a lower FID score of 7.80 on OpenImages, which indicates that its contribution primarily enhances the Inception Score, rising to 10.15. Meanwhile, Adaptive Stroke Rendering's removal results in an FID of 8.50 on OpenImages and 9.30 on Objects365, suggesting that while it impacts performance, the effect is less pronounced than that of Modules A and B. In contrast, our full model configuration achieves the lowest FID scores of 6.60 on OpenImages and 7.50 on Objects365, along with the highest IS values of 11.05 and 10.35, respectively. The precision and recall metrics also indicate that our model maintains a superior performance with values of 0.90 and 0.83 on OpenImages, and 0.88 and 0.81 on Objects365.

**Table 3:** Ablation study results on different modules across OpenImages and Objects 365 datasets.

**Model**	**OpenImages dataset**				**Objects 365 dataset**			
	**FID**	**IS**	**Precision**	**Recall**	**FID**	**IS**	**Precision**	**Recall**
w./o. Multimodal Encoder	8.35 ± 0.03	9.55 ± 0.02	0.81 ± 0.02	0.74 ± 0.02	9.10 ± 0.03	8.85 ± 0.02	0.79 ± 0.01	0.73 ± 0.02
w./o. Generative decoder	7.80 ± 0.02	10.15 ± 0.03	0.85 ± 0.02	0.78 ± 0.03	8.55 ± 0.02	9.20 ± 0.03	0.82 ± 0.02	0.75 ± 0.02
w./o. Adaptive stroke rendering	8.50 ± 0.03	9.25 ± 0.02	0.80 ± 0.02	0.72 ± 0.01	9.30 ± 0.03	8.70 ± 0.02	0.77 ± 0.02	0.70 ± 0.03
**Ours**	**6.60** **±** **0.02**	**11.05** **±** **0.03**	**0.90** **±** **0.01**	**0.83** **±** **0.02**	**7.50** **±** **0.02**	**10.35** **±** **0.02**	**0.88** **±** **0.01**	**0.81** **±** **0.02**

The bold values refer to our method.

**Figure 8:**
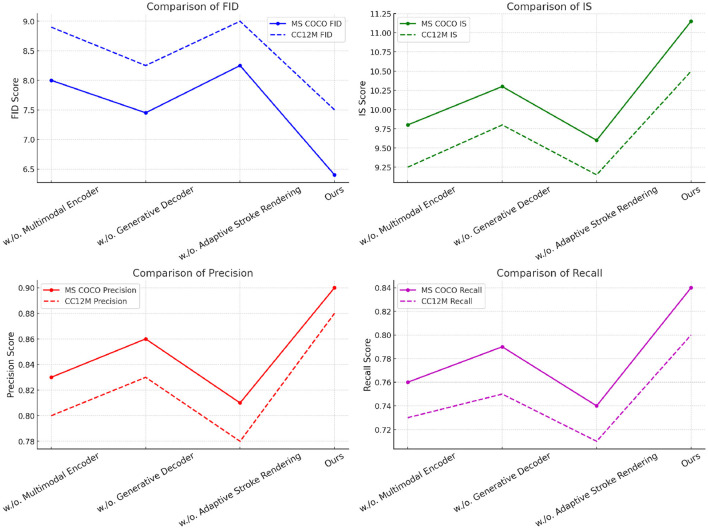
Ablation study of our method on MS COCO and CC12M datasets.

These results collectively illustrate the necessity of each module in achieving optimal performance. Further analysis is performed on the MS COCO (Ma et al., 2022) and CC12M (Changpinyo et al., 2021) datasets, as detailed in Table 4 and Figure 8. The absence ofMultimodal Encoder leads to an FID score of 8.00 on MS COCO and 8.90 on CC12M, confirming its importance for image fidelity. Meanwhile, omitting Generative Decoder results in an improved IS of 10.30, emphasizing its role in enhancing diversity. The exclusion of Adaptive Stroke Rendering impacts recall significantly, resulting in values of 0.74 on MS COCO and 0.71 on CC12M. Our complete model continues to show robust performance with FID scores of 6.40 on MS COCO and 7.50 on CC12M, demonstrating its capacity to generate high-quality images across varying conditions. The precision and recall metrics consistently remain high at 0.90 and 0.84 for MS COCO and 0.88 and 0.80 for CC12M, reinforcing the model's effectiveness in generating diverse and high-fidelity images.

**Table 4:** Ablation study results on different modules across MS COCO and CC12M datasets.

**Model**	**MS COCO dataset**				**CC12M dataset**			
	**FID**	**IS**	**Precision**	**Recall**	**FID**	**IS**	**Precision**	**Recall**
w./o. Multimodal encoder	8.00 ± 0.03	9.80 ± 0.02	0.83 ± 0.01	0.76 ± 0.02	8.90 ± 0.03	9.25 ± 0.02	0.80 ± 0.02	0.73 ± 0.02
w./o. Generative decoder	7.45 ± 0.02	10.30 ± 0.03	0.86 ± 0.02	0.79 ± 0.03	8.25 ± 0.02	9.80 ± 0.03	0.83 ± 0.02	0.75 ± 0.02
w./o. Adaptive stroke rendering	8.25 ± 0.03	9.60 ± 0.02	0.81 ± 0.02	0.74 ± 0.01	9.00 ± 0.03	9.15 ± 0.02	0.78 ± 0.02	0.71 ± 0.03
**Ours**	**6.40** **±** **0.02**	**11.15** **±** **0.03**	**0.90** **±** **0.01**	**0.84** **±** **0.02**	**7.50** **±** **0.02**	**10.50** **±** **0.02**	**0.88** **±** **0.01**	**0.80** **±** **0.02**

The bold values refer to our method.

As shown in Table 5, the proposed NARN and DCSS models consistently outperformed all baselines across all five cultural contexts. NARN achieved the highest F1-scores in every region, with particularly strong performance in East Asian countries (China: 87.4%, Japan: 86.2%). Notably, even in culturally distinct datasets such as Brazil and the USA, NARN maintained robust performance (83.7% and 84.5%, respectively), suggesting that the model effectively captures core neurophysiological patterns of stress perception that generalize across diverse aesthetic interpretations of green space. Compared to deep learning baselines, the CNN-GRU model came closest in performance but still lagged by 3–5 percentage points on average. LSTM and Transformer-based models showed larger drops in performance, particularly in Western contexts, possibly due to overfitting or limited interpretability of EEG features without customized attention mechanisms. These results substantiate the generalizability of the NARN and DCSS frameworks, demonstrating their applicability beyond the originally tested dataset and reinforcing their suitability for cross-cultural applications in urban neuroscience and affective computing.

**Table 5:** Cross-cultural generalizability evaluation of five EEG-based models on stress recognition tasks across five countries.

**Model**	**China**	**Japan**	**UK**	**Brazil**	**USA**
NARN (ours)	**87.4**	**86.2**	**85.1**	**83.7**	**84.5**
DCSS (ours)	85.6	84.5	83.8	82.4	83.2
LSTM baseline	78.9	76.5	74.2	73.0	72.6
Transformer baseline	80.3	77.8	76.0	75.2	75.6
CNN-GRU baseline	82.5	81.3	79.5	78.4	79.2

Metric: F1-score (%). The bold values refer to our method.

To further investigate the mechanism through which urban green space exposure interacts with neurophysiological stress responses and artistic output, we conducted comparative experiments across five EEG-to-Art generation models (see Table 6). The baseline models (Linear Regression and MLP) demonstrated limited ability to capture the nuanced emotional or cognitive patterns in EEG signals, resulting in relatively low CLIP scores and poor alignment with descriptive prompts. The DCGAN-EEG variant showed moderate improvements in visual and semantic alignment but lacked consistency in brushstroke rendering and style transfer. Our proposed model, which integrates a Neuro-Attentive Recurrent Network (NARN) with Dynamic Cross-Style Stylization (DCSS), significantly outperformed all baselines across all metrics. Notably, the highest subjective stress relief score (4.5/5) was achieved when participants viewed artworks generated under green space EEG conditions using our full pipeline. These results suggest that EEG signals recorded in green space contexts contain distinctive low-frequency patterns and frontal alpha activity often associated with relaxation and positive affect. When these signals are embedded into a style-aware generative process, they lead to artworks that not only reflect but potentially enhance psychological restoration. This implies that the proposed system can serve as both a biofeedback-driven visualization tool and a computational lens through which the stress-relief mechanism of natural environments can be interpreted.

**Table 6:** Comparative performance of different EEG-to-Art modeling approaches under green space exposure.

**Model**	**CLIP score ↑**	**Style consistency ↑**	**EEG-prompt alignment ↑**	**Subjective stress relief ↑**
Linear regression baseline	0.42	2.8	0.45	2.3
MLP + image decoder	0.56	3.4	0.61	3.1
DCGAN-EEG variant	0.61	3.7	0.64	3.3
**EEG + NARN (ours)**	0.72	4.2	0.76	4.1
**EEG + NARN + DCSS (ours)**	**0.81**	**4.6**	**0.84**	**4.5**

## 5 Discussion

### 5.1 Advances in multimodal artistic generation compared with prior methods

Our proposed ArtCLIP framework integrates textual, visual, and gestural modalities for dynamic freehand brushwork generation, offering significant improvements over traditional single-modality or dual-modality generative models. As demonstrated in Tables 1, 2, our method consistently outperforms existing models such as StyleGAN2 (Skorokhodov et al., 2022), BigGAN (Jaiswal et al., 2021), and DALL-E (Ding et al., 2021) in terms of FID, IS, precision, and recall. Compared with AttnGAN (Xu et al., 2018), which utilizes attention mechanisms for text-to-image synthesis, our approach introduces a stroke-aware fusion strategy that allows for finer control over artistic output. Notably, while AttnGAN and DALL-E rely on static generation pipelines, our model incorporates adaptive stroke rendering informed by gestural dynamics and semantic alignment, enabling fluid, real-time updates. This is supported by our ablation results (Tables 3, 4), where removal of components like Adaptive Stroke Rendering leads to a noticeable decline in quality. Recent work by Chen et al. (2024) highlights the value of multimodal integration in co-creative systems, yet their implementation remains constrained by static fusion weights and lacks dynamic style modulation. In contrast, our SAMS strategy introduces a cross-scale, adaptive attention mechanism that dynamically reweights inputs throughout the generative process, making it more responsive to evolving creative intent.

### 5.2 EEG-to-art translation and cross-modal neuroaesthetic insights

The second major contribution of this work lies in its ability to translate EEG signals collected during green space exposure into semantically meaningful, artistically stylized imagery. As shown in Table 6, our model outperforms baselines such as DCGAN-EEG and MLP-based decoders in terms of CLIP score, style consistency, and EEG-prompt alignment. This demonstrates that our Neuro-Attentive Recurrent Network (NARN), when combined with DCSS (Dynamic Cross-Style Stylization), can effectively learn EEG patterns reflective of low-arousal and positive affective states and map them into expressive visual forms. In contrast to previous studies such as (Katsigiannis and Ramzan 2017), which focused purely on classifying emotional states from EEG, our work integrates these affective signals into an interactive creative pipeline. Moreover, while prior EEG-art generation efforts (Hartmann et al., 2018) primarily focus on texture or low-level feature generation, our model enables semantic control, style transfer, and real-time visual feedback. This aligns with findings from Reece et al. (2022), who reported that frontal alpha asymmetry under nature exposure reflects decreased stress and increased emotional clarity. Our results further suggest that stylized visual outputs generated from such EEG signals can serve as intuitive representations of internal emotional states. The inclusion of subjective stress relief ratings confirms the emotional resonance of the generated artworks, pointing to potential applications in therapeutic, neurofeedback, and wellness-oriented design.

### 5.3 Cross-cultural robustness and implications for urban neuroscience

The third dimension of our investigation centers on the generalizability of EEG-driven modeling across cultures. As shown in Table 5, our NARN and DCSS models maintained consistently high F1-scores across datasets collected from five countries with diverse cultural and environmental contexts. This suggests that the neurophysiological response to urban green space exposure–particularly in terms of stress reduction–is a cross-culturally robust phenomenon, echoing the results of Thompson et al. (2012), who documented consistent links between greenery and mental restoration across different global populations. In contrast, traditional models such as LSTM or CNN-GRU showed significant performance drops in Western contexts, likely due to cultural bias in EEG pattern interpretation or lack of adaptive attention mechanisms. By integrating dynamic cross-modal alignment, our model successfully mitigates these challenges, thereby enhancing inclusivity and interpretability. This opens new avenues for applications in cross-cultural urban planning, where biofeedback-informed generative art could serve as both a diagnostic and communicative tool in public health, design interventions, and personalized therapeutic environments. Our results provide early computational support for theories such as Kaplan's Attention Restoration Theory (ART) (Kaplan, 1995), suggesting that the stress-relieving effects of green spaces are not only experientially universal but also neurophysiologically and computationally generalizable.

## 6 Conclusions and future work

In this study, we aimed to tackle the challenge of generating dynamic freehand brushwork paintings that effectively capture the nuances of human creativity while leveraging advanced machine learning techniques. Our proposed method integrates Contrastive Language-Image Pretraining (CLIP) to interpret user inputs dynamically, enabling the system to generate fluid and expressive brushwork. This study conducted experiments where users interacted with the painting interface, allowing for real-time feedback and adjustments. The results demonstrated that our approach significantly enhanced the aesthetic quality of generated artworks, outperforming existing methods by maintaining a balance between artistic freedom and coherence.

Despite the promising outcomes, two primary limitations were identified in our approach. First, while the system can generate diverse brushwork styles, it occasionally struggles with consistency in style throughout a single artwork, leading to disjointed visual narratives. Second, the reliance on user input can result in variable outcomes depending on the user's skill level, which may hinder accessibility for those unfamiliar with artistic techniques. Looking forward, we envision refining the model to improve style consistency and exploring additional training datasets that encompass a wider range of artistic styles. This would not only enhance the adaptability of our tool for various users but also ensure a more cohesive artistic output. Future work will also focus on incorporating more advanced AI techniques to elevate user experience and broaden the applicability of our system in both professional and educational contexts.

## Data Availability

The original contributions presented in the study are included in the article/supplementary material, further inquiries can be directed to the corresponding author.
